# The Not4 RING E3 Ligase: A Relevant Player in Cotranslational Quality Control

**DOI:** 10.1155/2013/548359

**Published:** 2013-01-21

**Authors:** Martine A. Collart

**Affiliations:** Department of Microbiology and Molecular Medicine, Faculty of Medicine, CMU, University of Geneva, 1 Rue Michel Servet, 1211 Geneva 4, Switzerland

## Abstract

The Not4 RING E3 ligase is a subunit of the evolutionarily conserved Ccr4-Not complex. Originally identified in yeast by mutations that increase transcription, it was subsequently defined as an ubiquitin ligase. Substrates for this ligase were characterized in yeast and in metazoans. Interestingly, some substrates for this ligase are targeted for polyubiquitination and degradation, while others instead are stable monoubiquitinated proteins. The former are mostly involved in transcription, while the latter are a ribosomal protein and a ribosome-associated chaperone. Consistently, Not4 and all other subunits of the Ccr4-Not complex are present in translating ribosomes. An important function for Not4 in cotranslational quality control has emerged. In the absence of Not4, the total level of polysomes is reduced. In addition, translationally arrested polypeptides, aggregated proteins, and polyubiquitinated proteins accumulate. Its role in quality control is likely to be related on one hand to its importance for the functional assembly of the proteasome and on the other hand to its association with the RNA degradation machines. Not4 is in an ideal position to signal to degradation mRNAs whose translation has been aborted, and this defines Not4 as a key player in the quality control of newly synthesized proteins.

## 1. Introduction 

The appropriate control of gene expression is essential to the development and growth of all organisms. Ultimately, gene expression is the production of functional proteins at the appropriate time and level, in their appropriate cellular localization and state for interaction with their correct physiological partners. Many things can go wrong between transcription of a gene and this ultimate goal. Sophisticated surveillance mechanisms have therefore evolved to follow gene expression at every step and destroy aberrant products whose accumulation can be toxic, leading ultimately to cell death. 

Proteins mediate almost all cellular functions. Hence one crucial step in the expression of a gene is the synthesis of its encoded polypeptide at the ribosome. This process involves many interactions, constraints, modifications, and precisely defined kinetics of synthesis. Multiple quality control systems ensure that the newly synthesized proteins ultimately achieve their native functional form, or if they do not, they get removed and destroyed. This paper will summarize the different components that contribute to this quality control system and present a new and relevant player, the Not4 RING E3 ligase. 

## 2. Folding of Newly Synthesized Polypeptides

 Proteins must adopt their appropriate folded state to be functional, and the folding of a protein is dictated by its primary amino acid sequence [1, 2]. A protein is often composed of separate domains that can fold independently, but proteins must also adopt precise three dimensional conformations to be fully active. In the cell, despite the fact that the linear sequence of a polypeptide chain contains all of the necessary information to specify the three-dimensional structure, its folding is aided by molecular chaperones [3]. This ensures that proteins fold efficiently with a time scale that will allow them to perform their biological function. Protein folding is widely believed to start during synthesis at the ribosome, in other words cotranslationally [4–6]. Immediately after synthesis is initiated, as the nascent chain exits from the ribosome tunnel, it undergoes various modifications and is subject to interactions with ribosome-associated chaperones. These chaperones can function as folding catalysts and/or targeting molecules. 

**Figure 1:**
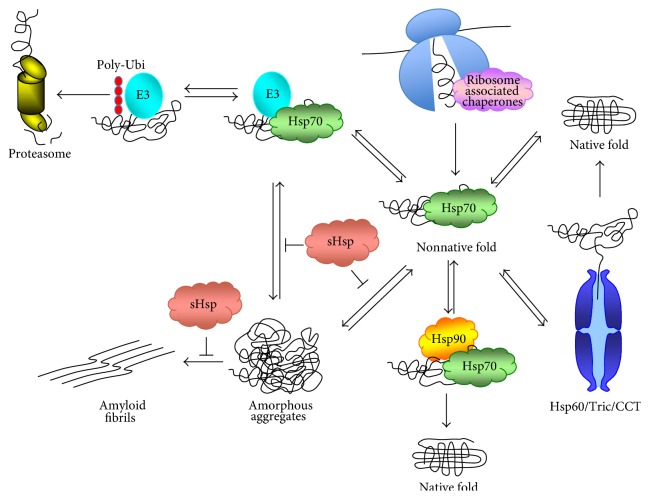
Role of the molecular chaperones in protein folding, removal from aggregates, and delivery to the proteasome. Newly synthesized proteins start to fold cotranslationally, and they are assisted by ribosome-associated chaperones. Not all proteins reach their native state after synthesis, and they are assisted by ATP-dependent chaperones of the Hsp70 family. They will then either fold into their native state or require yet other chaperones such as Hsp60 or Hsp90. Some proteins might not fold and instead aggregate. They can subsequently be refolded or delivered to the UPS system, in both cases with the help of molecular chaperones. Alternatively they might form toxic amyloid fibrils (figure adapted from [14]).

In eukaryotes, proteins will not always be completely folded at the end of synthesis. After intervention of ribosome-associated chaperones, some proteins will need additional assistance from subsequent chaperones, with a well-defined sequence of chaperone interactions (for review see [7, 8]). First the ATP-dependent molecular chaperones of the Hsp70 family that do not bind directly the ribosome (such as the Ssa1-4 proteins in yeast) come into action (Figure 1). They prevent aggregation by shielding hydrophobic segments, until they can release fast-folding molecules. In addition to assisting posttranslational folding, these chaperones participate in various other functions such as assisting proteolytic degradation of aggregated proteins and protein trafficking. Hsp70 chaperones can deliver substrates that do not reach fast-folding states to chaperonins, chaperones of the Hsp60 family (such as TRiC/CCT in yeast), large double-ring complexes that promote folding through ATP-dependent cycles of global protein encapsulation, one molecule at a time (for review see [9]). About 10% of newly synthesized cytosolic proteins interact with TRiC. It has been suggested that TRiC can assist cotranslational folding of individual domains of larger proteins because it can interact with nascent chains [10, 11]. Hsp90 is a one more chaperone system that can function downstream of the Hsp70s. It cooperates with a multitude of regulators and co-chaperones, and hence is a rather complex system (for review see [12]). It participates in the final maturation of many transcription factors and signaling molecules and hence to a variety of cellular processes. Finally, small heat shock proteins (called sHSPs, such as Hsp26 and Hsp42 in yeast) suppress amorphous aggregates of denatured proteins [13]. They are the most widespread family of molecular chaperones and protect cells from environmental stresses. All of these chaperones participate to prevent protein misfolding both under normal conditions and under stress conditions when the concentration of improperly folded proteins increases (Figure 1). 

Ultimately proteins will fold into their native form. If not, the chaperones will also participate in the delivery of the misfolded proteins not only to the protein degradation machinery, mostly to the ubiquitin proteasome system (UPS), but also to autophagy (for review see [17]). Proteins to be degraded by the UPS must be marked by the covalent attachment of a polyubiquitin chain, which is then recognized by the 26S proteasome. Ubiquitination of proteins occurs in several steps and is catalyzed by 3 classes of enzymes called ubiquitin-activating enzyme (E1), ubiquitin-conjugating enzymes (E2s, represented by a small group of proteins), and finally ubiquitin-ligating enzymes (E3s), which determine the substrate specificity and which are very diverse.

Proteins that do not finally fold after synthesis, or proteins that become unfolded because of stress, can accumulate in amorphous aggregated forms. This can serve as a temporary deposit from which chaperones will either resolubilize proteins at a later stage or deliver them to the degradation machines (Figure 1). In yeast distinct compartments containing misfolded proteins have been characterized according to the mobility and ubiquitination state of aggregated proteins. These are JUNQ (juxta nuclear quality control compartment), which is mobile, ubiquitinated, and juxta nuclear, IPOD (insoluble protein deposit), immobile and nonubiquitinated [18], additional compartments that interact with the small heat shock protein Hsp42 in a more peripheral localization [19], and finally aggresomes that are associated with microtubules [20]. A network of E3 ligases, in combination with molecular chaperones, act to prevent the excessive accumulation of aggregated proteins or to clear them once formed [21]. The precise role of the chaperones in the clearance of misfolded proteins has not been definitely established. On one hand they certainly function to stabilize and refold the nonnative polypeptides, but on the other hand, they interact with the E3 ligases and might function to recruit these enzymes to misfolded proteins (Figure 1) [22, 23]. Proteins in aggregates, instead of being resolubilized or degraded, can adopt toxic configurations such as the amyloid like aggregates seen in many neurodegenerative disorders [24] (Figure 1). 

## 3. Interactions at the Vicinity or Just outside of the Tunnel Exit

**Figure 2:**
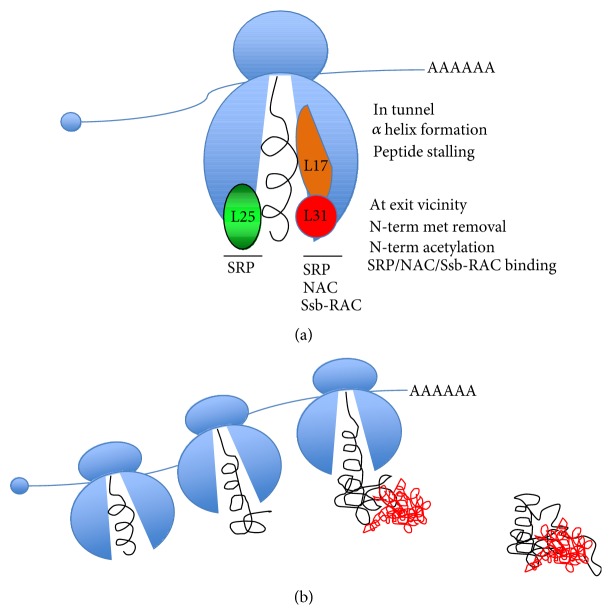
(a) Cotranslational folding, modification, and interaction with ribosome-associated chaperones. The large ribosomal subunits that interact with the ribosome-associated chaperones NAC, Ssb/RAC, and SRP (themselves not depicted) are indicated in color (L17 stands for Rpl17, L25 stands for Rpl25, and L31 stands for Rpl31; figure adapted from [15]). (b) Cotranslational assembly of cellular complexes. A newly synthesized protein (in black) associates with its partner protein (in red) already during its synthesis (figure adapted from [16]).

Synthesis of proteins starts at the peptidyl transferase center of the ribosome from where the newly synthesized polypeptide will start traveling through the ribosome exit tunnel of the large ribosomal subunit in a vectorial manner (for review see [25]). This tunnel is composed primarily of the largest mature rRNA and the eukaryotic L4, L17, and L25, large ribosomal subunits, the latter comprising in large part the exit site [26] (Figure 2(a)). The tunnel is not inert, but seems to participate in nascent chain folding [27–29]. 

As soon as the nascent polypeptide leaves the ribosome tunnel, it will interact in the vicinity of the tunnel with modifying enzymes and chaperones [5]. Many cotranslational protein modifications can occur, such as removal of the N-terminal methionine [30, 31], N-terminal acetylation [32, 33], N-terminal myristoylation [34, 35], N-linked glycosylation [36, 37], cotranslational peptide cleavage [38], and disulfide bond formation, reduction, or isomerization [39]. These modifications are obviously essential for proteins to reach their fully functional active state, but they will not be discussed further in this paper. Suffice it to say that the enzymes will need to have access to the emerging peptide at the appropriate time and in the context of the chaperones present at the tunnel exit.

 The chaperone partner for the emerging nascent chain will be mostly defined by the final cellular destination of the newly synthesized protein. Nascent chains of cytosolic proteins interact with ribosome-associated chaperones (for review see [40]). In the yeast *S. cerevisiae*, there are 2 distinct ribosome-associated chaperones: the nascent associated polypeptide complex (NAC) and the Ssb/Ssz/zuotin triad (called Ssb/RAC), conserved in other eukaryotes. Instead, for secreted proteins and most membrane proteins, the nascent chain is mostly hydrophobic in sequence and will interact with the signal recognition particle (SRP) [41–43].

### 3.1. NAC

NAC is the first protein thought to interact with the emerging nascent chain during translation (for review see [44]). It is an *α*/*β* heterodimer originally described to interact with the L23 subunit of the *E. Coli* ribosome (equivalent to L25 in eukaryotic ribosomes) through its *β* subunit [45]. A motif in the *β* subunit, RRK(X)nKK, between amino acids 24 and 30 was identified as necessary and sufficient to target NAC to the ribosome. More recently a different binding site for NAC on the ribosome was described. The N-terminal 23 amino acids of NAC*β* bind near the tunnel exit protein Rpl31, while NAC*α* interacts with its neighbor protein Rpl17 at the ribosome tunnel exit [46] (Figure 2). NAC is thought to function also as a homodimer, in particular as a *β*/*β* homodimer [47, 48]. In yeast, 2 genes encode the *β* NAC subunit, *BTT1* and *EGD1*, while only one, *EGD2*, encodes the *α* subunit. The 2 subunits show substantial homology to each other [49], but the NAC*α* subunit has an N-terminal UBA domain that is absent in NAC*β* [50]. Both NAC subunits are ubiquitinated, and their ubiquitination is interdependent: that of NAC*β* depends upon NAC*α*, while that of NAC*α* increases in the absence of NAC*β* ubiquitination [51].

Several roles have been attributed to NAC, including protection of the emerging nascent chain from proteolysis or from interaction with the cytosol [52] or participation in the initial folding of the polypeptide [53]. NAC has also been suggested to regulate access of the ribosome to the translocation pore of the ER membrane during cotranslational targeting of nascent proteins to the ER [53–55]. Analysis by microarrays of the NAC-associated translatome in yeast [48] has revealed that NAC is a general cotranslational chaperone, associating with polysomes translating all mRNAs. However the different NAC dimers are targeted to polysomes translating specific classes of mRNAs. Btt1 homodimers are enriched in ribosomes translating ribosomal proteins or mitochondrial proteins while the Egd1/Egd2 heterodimers show some specificity towards ribosomes translating metabolic enzymes. It is intriguing that Btt1 is enriched in ribosomes translating mitochondrial proteins, because early studies have suggested that NAC plays a role in the attachment of cytosolic ribosomes to mitochondria [56, 57]. 

At this time, we still do not understand how the specificity of chaperone association with polysomes is dictated *in vivo*, and the function of NAC is still largely debated. NAC is not essential in yeast, but its deletion leads to embryonic lethality in higher eukaryotes. In yeast, NAC deletion starts to show growth phenotypes in the absence of the other ribosome-associated chaperone, Ssb/RAC [58], suggesting that the 2 ribosome-associated chaperone systems may be partially redundant [59]. 

### 3.2. Ssb/RAC

This second ribosome-associated chaperone is composed of 2 Hsp70 family members, Ssb (encoded by 2 genes in yeast, *SSB1* and *SSB2*) [60] and Ssz (encoded by *SSZ1*) [61]. Zuotin (encoded by *ZUO1*) [62] is a J-domain Hsp40 co-chaperone for Ssz, and together they stimulate the ATPase activity of Ssb. Zuo1 brings RAC to the ribosomal protein L31, close to the ribosome tunnel exit site [15]. Ssb seems to be the component of this chaperone that can interact with the emerging nascent chain. RAC/Ssb is thought to be involved in the folding and assembly of ribosomal proteins [59, 63].

### 3.3. SRP

SRP is a universally conserved ribonucleoprotein complex (RNP-complex). In eukaryotes, SRP contains 6 distinct proteins and a 7S RNA [41–43]. On the ribosome SRP interacts with L25 [64], independently of an emerging nascent chain [65], but in a position to interact with a target nascent chain and to accelerate targeting of the ribosome nascent chain complex to the translocon [41–43]. Once SRP is associated with a target nascent chain, translation undergoes transient arrest. This enables SRP to associate with its receptor (SR) on the translocation machinery associated with the ER membrane, in order to favor translocation of the newly synthesized polypeptide into the ER. Interestingly, structure analysis of the docking complex composed of SRP, SR, and the translating ribosome revealed that Rpl31 was the contact site of the SR [66]. Thus, NAC and SR might be in competition for the same universal Rpl31/Rpl17 binding site on the ribosome (Figure 2(a)), and consistently it was shown that NAC modulates the specificity of SRP's association with translating ribosomes [48]. This probably explains NAC's influence on targeting the ribosome nascent chain complex to the ER translocon. Despite the fact that the association of Ssb/RAC has also been mapped to L31, it seems to be slightly different and may not be in competition with SRP [15].

### 3.4. Cotranslational Complex Assembly

 A recent study by Duncan and Mata have provided evidence for widespread cotranslational assembly of multi-subunit complexes [16]. Using a sample of proteins that do not contain RNA-binding domains, they determined that nearly 40% of them were associated with mRNAs that encode interacting proteins, in a manner that was dependent upon polysome integrity. The model emerging from these findings is that complexes start to be assembled cotranslationally (Figure 2(b)). Very little is known about this process; namely, how proteins are present at the correct polysomes, and how they are brought to the emerging nascent peptide. One can imagine that a protein will associate with its partner protein when the domain that it recognizes folds. How these events are coupled to ribosome-associated chaperones recognizing the nascent peptide and cotranslational protein modifications remains to be investigated. In any event, one must conclude from these findings that inefficient cotranslational complex assembly is likely to induce quality control mechanisms. 

## 4. Cotranslational Quality Control

As mentioned above, nascent polypeptides start to fold immediately upon synthesis and gradually reach more complex folding structures as they emerge from the ribosome and integrate protein modifications and new interactions. Ultimately they should reach their native state. However, despite the myriad of players present to ensure that a protein reaches its native state, mis-folding can occur. Consequently, cells have developed sophisticated systems to control the quality of newly synthesized proteins and degrade misfolded proteins cotranslationally [67, 68]. The extent of this is not exactly known. It was initially claimed that up to 30% of newly synthesized proteins are degraded [67], but this was probably an overestimation due to the experimental conditions used [69], since stressful environmental conditions can increase misfolding.

### 4.1. Expected Causes for Induction of a Quality Control Response

Misfolding of nascent proteins synthesized from normal mRNAs can result from kinetics of translation that do not allow domains to fold as they emerge from the ribosomal tunnel. The presence of rare codons or amino acid shortage, for instance, can affect kinetics of translation. Misfolding can also result from insufficient chaperone capacity or absence of interacting partners. Problems in folding can stem from aberrant mRNAs carrying missense mutations. In addition a ribosome may stall for a number of reasons. These include specific stable structures of the mRNA such as stem loops or damaged RNA bases, protein sequences encoded by specific clusters of codons, or because the mRNAs encode proteins interrupted prematurely by a stop codon. Finally a quality control response may occur when mRNAs lack a stop codon for the encoded protein. 

Hence, cotranslational quality control will occur whether a problem is encountered at the level of the folding of the nascent polypeptide, stalling of the ribosome, or if the mRNA is aberrant. It will require that the problem is sensed, ribosomes stalled, translation arrested, the protein disposed of, the mRNA degraded, and the ribosome released, in an order of events that may not always be the same depending upon the initial problem.

### 4.2. Translation Arrest

It is generally believed that sensing of a misfolded nascent polypeptide or aberrant mRNA undergoing translation will lead to a translation arrest and that this arrest induces the downstream events. In some cases it is thought that ribosome stalling might be the initial event. Nevertheless, ribosome stalling per se cannot be sufficient for induction of the quality control response. Indeed ribosome profiling experiments [70] have revealed that there are numerous pausing events during translation across the genome. Clearly pausing during translation of normal essential cellular proteins cannot lead to destruction of the newly synthesized peptides and their mRNAs. Maybe the duration of stalling is what distinguishes productive translation from abortive translation, or it is maybe the combination of stalling with additional features of the newly synthesized peptide or of the mRNA that is relevant. 

### 4.3. mRNA Surveillance

 Significant knowledge of how features of mRNAs can be recognized as aberrant by the mRNA surveillance machinery and induce a cotranslational quality control response is available and was recently reviewed [71]. There are 3 pathways of cotranslational surveillance that have generally been distinguished. These are the nonsense mediated decay (NMD), the nonstop decay (NSD), or the no-go decay (NGD). 

**Figure 3:**
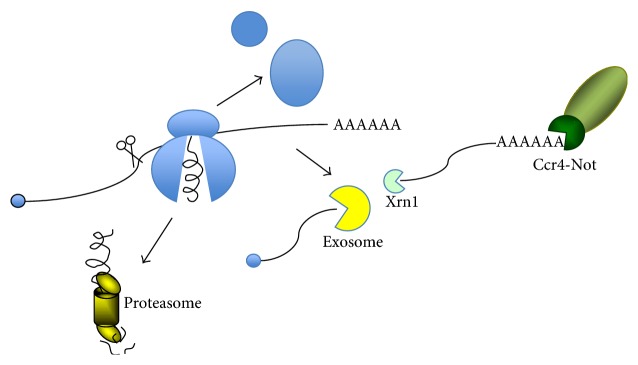
Common features of mRNA surveillance upon NDM, NGD, and NSD. After endonucleolytic cleavage upstream of a stalled ribosome, 3′ to 5′ degradation by the exosome, 5′ to 3′ degradation by the Xrn1 exonuclease, and deadenylation by the Ccr4-Not complex will occur. In all cases the ribosome will be released and the translation product degraded by the proteasome (adapted from [71]).

NMD occurs if a stop codon is encountered prematurely. It is the most well-studied mRNA surveillance mechanism and many reviews have been written on different aspects of NMD (see [72] and references therein). Authentic stop codons are localized at the 3′ end of mRNAs and are recognized by the translation termination factors eRF1 and eRF3. Premature stop codons (PTCs) are also recognized by eRF1 and eRF3. The Upf factors (Upf1, Upf2, and Upf3), identified originally by genetic selections in yeast [73, 74], are the key factors involved in recognizing these stop codons as premature. Upf1, an ATPase and helicase, interacts with eRF1/3 on one hand and with Upf2/3 on the other. Somehow these factors serve as a scaffold for other factors important for NMD. The exact mechanism whereby a codon is recognized as premature is unclear. In higher eukaryotes, the fact that it may reside upstream of an exon junction complex (EJC), while usual stop codons are present in the most 3′ exon, is thought to contribute to NMD. However, NMD occurs in yeast, where introns are rare, suggesting that the EJC cannot be the essential component of NMD [75]. Several alternative models have been proposed, such as an interaction between Upf1 and the 3′ end of mRNAs, that would be sensitive to the distance to the end of the message [76], or Upf1 evaluation of encounters with termination codons in the decoding center [77]. Upon NMD, an endonucleolytic event occurs upstream of the stalled ribosome, and then mRNAs undergo accelerated decay by the Xrn1 exonuclease from the 5′ end for the mRNA segment downstream of the cleavage and from the 3′ end via recruitment of the exosome by Ski7 for the upstream mRNA segment [78, 79] (Figure 3). This upstream mRNA produced by cleavage is in essence an mRNA lacking a stop codon and hence probably degraded accordingly (see below). It is thought that Upf1 associates with (and probably recruits) many of the RNA degradation factors. These include, for instance, the Smg5/6/7 proteins required for endonucleolytic cleavage and decay of PTC containing nonsense mRNAs in mammalian and *Drosophila* cells. Smg6 has a PIN domain and is the endonuclease that cleaves the non-sense mRNA upstream of the arrested ribosome [79–81]. No similar endonucleolytic event has been demonstrated in yeast upon NMD.

NGD concerns mRNAs, which have sequences that will lead the translating ribosomes to stall. These can be not only specific mRNA structures such as stem loops, but also given peptide sequences or codon stretches [86–88]. These RNAs also undergo endonucleolytic cleavage upstream of ribosome stalling, but the endonuclease that participates needs to be defined. One protein, Asc1, has been implicated in the translation arrest and mRNA degradation resulting from synthesis of consecutive positively charged amino acids [89]. This protein, also called receptor for activated kinase C 1 (RACK1), is highly conserved in eukaryotes [90]. It is a core component of the 40S ribosomal subunit [91, 92] that has been implicated in Src-kinase and protein C kinase signaling pathways [93, 94]. It binds to the 40S near the mRNA exit channel and has been connected to the regulation of translation initiation [95, 96]. It seems to have an impact on the structure of the 60S in 80S ribosomes [95]. Asc1 is not indispensable for NGD, because its deletion does not entirely abolish production of truncated protein and mRNA. Two additional proteins, Dom34 and Hbs1, with structures reminiscent of the eRF1/3 termination factors [97–100], have been genetically connected to NGD [86]. They interact with the aminoacyl(A) site in a codon-independent manner, and they are thought to participate in an altered termination event that leads to ribosome dissociation [101]. They function most efficiently with very short sequences 3′ of the ribosome [102], a limitation imposed by the N-terminal region of Hbs1 that is located at the mRNA entry channel of the ribosome [103], and can monitor mRNA length. The endonucleolytic cleavage itself increases in the presence of Dom34 [86], suggesting that Dom34 has functions prior to ribosome recycling. Ribosomal subunit dissociation and probably recycling, be it that resulting from eRF1 or Dom34, is aided by Rli1, an essential ATPase, thought to force Dom34 or eRF1 through the ribosomal subunit interface [104–106]. Ski7 closely resembles the Hbs1 and eRF3 GTPases, [107], but no factor homologous to Dom34 or eRF1 has been identified as a partner for Ski7. 

NSD occurs when mRNAs lack an in-frame stop codon [108]. NSD occurs whether the mRNA is truncated and ribosomes simply run to the end, or whether the mRNA has its usual 3′ poly(A) sequence, in which case stalling has some aspects common to NGD, since translating poly(A) sequences will create a positively charged peptide that can interact with the negatively charged exit channel and cause translational arrest. When a ribosome runs to the very end of a nonstop transcript, Ski7 binds to the empty aminoacyl(A) site and recruits the exosome [109]. The Rrp44 catalytic subunit of the exosome then mediates not only 3′ to 5′ exonucleolytic degradation but also endonucleolytic cleavage of the mRNAs. Following endonucleolytic cleavage, a secondary NSD mRNA without poly(A) tail is created upstream. The RAC/Ssb chaperone has been implicated in maintaining low levels of translation of polylysine containing transcripts by contributing to translational repression during NSD [110]. This translational repression correlates with increased stability of the complex between the nonstop or C-terminally tagged polylysine proteins and the ribosome. Removal of the poly(A)-binding protein Pab1 by the ribosome upon NSD might also contribute to translation repression [111]. The role of RAC/Ssb in nonstop mRNA surveillance is consistent with previous experiments that have shown that loss of RAC/Ssb enhances reading through stop codons, whereas overexpression of Ssb allows their efficient recognition [112, 113]. RAC/Ssb affects the maintenance of the prion present in many laboratory yeast strains and which is a nonfunctional form of the translation termination factor eRF3 (Sup35) called (PSI+) [113–115]. 

To summarize, a common feature in all of these cases of mRNA surveillance is the stalling of the ribosome, followed by an endonucleolytic cleavage upstream of the stalled ribosome creating NSD mRNAs upstream and uncapped mRNAs downstream, which can then be degraded, respectively, 3′ to 5′ by the exosome and 5′ to 3′ by the Xrn1 exonuclease (Figure 3). The activity of Xrn1 in this case bypasses the rate-limiting deadenylation and decapping steps, even though deadenylation by the Ccr4-Not complex is still likely to occur. In all cases the ribosome from the mRNAs undergoing degradation will need to be released and the translation product degraded.

### 4.4. Protein Degradation

The truncated proteins arising from the stalled ribosomes in the various mRNA surveillance pathways described above are for the most part unlikely to be active and potentially deleterious. Hence they will need to be degraded. This is also the case for misfolded nascent proteins arising from altered translation kinetics or reduced chaperone activity. Many studies have identified these nascent peptides as targets for degradation by the proteasome [116–118]. In contrast, translation products arising from nonstop mRNAs but destined to organelles become halted in the ER membrane translocator and are released into the ER lumen by Dom34/Hbs1 or cleared from the Tom40 mitochondrial translocator by Dom34/Hbs1 [119]. 

Nevertheless, for cytosolic proteins, they need to be marked for cotranslational degradation. N-terminal acetylation has been indicated as a potential mark for degradation [120]. However most nascent proteins to be degraded are marked by ubiquitination [67, 68]. This raises the issue of which enzyme(s) ubiquitinates the misfolded or truncated newly synthesized proteins. 

### 4.5. Ltn1

Ltn1 is a RING E3 ligase that associates with 60S ribosomes and is identified as the ligase involved in cotranslational ubiquitination of nonproductive intermediates [121]. Cells lacking Ltn1 are specifically sensitive to hygromycin B, an antibiotic that affects translational fidelity and increases read through stop codons. They are also sensitive to mutations in eRF3 that lead to read-through of normal stop codons. The RING domain of Ltn1 as well as the capacity of Ltn1 to interact with E2 enzymes is required for this function of Ltn1 in quality control.

Ltn1 is the yeast homolog of Listerin, a protein reported to cause neurodegeneration in mice [122]. In yeast Ltn1 was identified in a screen for proteins that contribute to reduce expression of proteins encoded by nonstop mRNAs, but not to the expression of the related proteins whose translation was normally terminated by a stop codon [118]. It was also described as a proteasome-interacting protein [123]. Ltn1 does not reduce proteasome function in a general way nor is it generally important for degradation of unstable proteins. Instead, Ltn1 interacts with nonstop proteins specifically and is required for their ubiquitination and degradation. Accumulation of nonproductive translation products is synthetically enhanced by the deletion of Ltn1, the deletion of Ski7, and the deletion of Zuo1, consistent with the observation that the 3 proteins act at different levels: Ltn1 ubiquitinates the aberrant proteins, Ski7 recruits the exosome to degrade the aberrant mRNAs, and Ssb/RAC arrests translation. 

The model is that Ltn1 ubiquitinates the nascent proteins on stalled ribosomes and promotes dissociation of the ribosome, and this then leads to degradation by the proteasome. How Ltn1 is recruited to stalled ribosomes, and when it is recruited; namely, whether this occurs just prior to, or after, ribosome dissociation, and its relationship to Asc1, Dom34, and Hbs1, are open questions.

Quality control is by far not complete after the action of Ltn1. Indeed, the proteasome must be recruited to the ubiquitinated nascent chain, or the ubiquitinated nascent chain must be released and targeted to the proteasome, and the mRNA must be degraded, when this has not specifically been activated by mRNA surveillance. This is where the Not4 RING E3 ligase most likely comes into action.

## 5. Not4 a New Player in Cotranslational Quality Control

Not4 was first identified in several genetic screens in yeast (for review see [124]) from which it obtained several different names: *NOT4*, *SIG1*, and *MOT2 *[125–127]. Two of the screens were related to signaling through G-proteins, but the third screen was unrelated. Hence the genetic isolation of Not4 was not revealing to the true function of Not4. Nevertheless all 3 screens suggested a negative role for Not4 in gene expression. 

**Figure 4:**
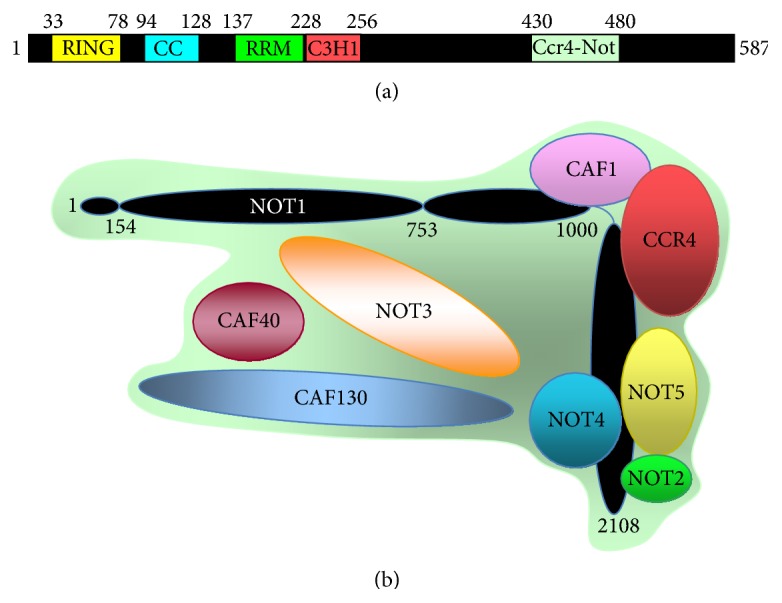
(a) Scheme of the *S. cerevisiae* Not4 protein. The different domains are indicated (RING: ring domain, CC: coiled coil, RRM, putative RNA recognition motif, C3H1 zinc finger, and Ccr4-Not interaction domain) [82]. (b) Scheme of the *S. cerevisiae* Ccr4-Not complex. The Ccr4-Not complex contains the 9 indicated proteins. Not2, Not4, and Not5 were mapped to the C-terminus of the Not1 scaffold by 2-hybrid experiments [83, 84], while a recent structure of Ccr4, Caf1, and Not1 confirmed the previous mapping to a central portion of Not1 [85]. The last 3 proteins have not been precisely positioned with regard to the scaffold.

The yeast Not4 protein is 587 amino acids long (Figure 4(a)) and is characterized by several domains [82]. In particular, it has in its N-terminus a RING finger domain [128] between amino acids 33 and 78, followed by a putative RNA binding domain between positions 137 and 228. In between these 2 domains, there is a putative coiled-coil region (amino acids 94–128). A second zinc finger of the C3H1 type is located further between amino acids 229 and 256. A clear function has been attributed only to the RING finger domain of Not4. This domain interacts with E2 enzymes [129], Ubc4, and Ubc5 in yeast [47, 130], and several mutations that abolish this interaction have been defined using the 2-hybrid assay [131]. These include L35A, I37A, and I64A, which have been subsequently used in functional studies. Other parts of the protein do not show recognizable motifs. 

Not4 is associated with 8 other yeast proteins in a large complex, called Ccr4-Not (for review see [132]) (Figure 4(b)). The C-terminal region of Not4 is required for interaction of Not4 with other Ccr4-Not subunits. In particular, the region between amino acids 430 and 480 contributes importantly to this interaction [82]. Not4 is associated with other Ccr4-Not subunits in a core complex of 0.9–1 MDa, but also in heterogenous other larger complexes, which elute with apparent sizes of about 2 MDa from gel filtration columns [83]. Besides ubiquitination provided by Not4, this complex contains another enzymatic activity, which is deadenylation carried by the Ccr4 and Caf1 subunits [133, 134]. Not4 together with Not2 and Not5 associates with the C-terminal region of the scaffold of the complex, Not1, whereas the deadenylase module interacts with a central domain of Not1 [83–85]. The position of the other 3 subunits is not well defined. The 2 enzymatic activities are located at different ends of the scaffold, but the 2 ends of the scaffold have been shown to come together when expressed in trans [83]. Besides the enzymatic subunits and the scaffold subunit, the role of the other subunits of the complex is not understood [132].

Not4 is also present in higher eukaryotes, where it interacts with Ccr4-Not orthologs [135–137]. However, in contrast to the situation in yeast, it is not a stable subunit of Ccr4-Not complexes in the other eukaryotes. Nevertheless, Not4 is functionally conserved. Indeed, the human subunit can complement the absence of the yeast subunit in a strain lacking another subunit of the Ccr4-Not complex, Not5, conditions in which yeast Not4 is essential [135]. 

In yeast, Not4 is not essential for viability. It is necessary for growth at high temperature, and cells lacking Not4 are sensitive to growth on different media, in particular media containing translation inhibitors such as cycloheximide (CHX) and hygromycin B or containing amino acid homologs such as azetidine-2-carboxylic acid, a proline analog (AZC) ([82, 138] and our unpublished observations) or finally on media containing DNA-damaging agents such as methyl methane sulfonate (MMS) or hydroxyurea (HU) [139, 140]. In contrast, cells lacking Not4 are resistant to heat stress [131]. The deletion of the RING finger of Not4 has essentially all of the same growth phenotypes as the complete deletion of Not4 [82] (Table 1). In contrast, point mutants in the RING domain of Not4 that abolish its interaction with its E2 partner enzymes do not have striking growth phenotypes, at either 30 or 37°C on rich medium [131]. They are sensitive to growth on media containing translational inhibitors, and they are resistant to heat stress like the complete deletion of Not4. This suggests that the RING domain of Not4 mediates most of the important functions of Not4, but that this is not always through its function as an E3 ligase. It could be that the RING domain of Not4 does not only function directly in ubiquitination but also as an interaction domain for other proteins. This still has to be clarified. It is curious to note that in a genome-wide search for genes which become essential when Not4 is deleted or mutated, more genes were recovered for synthetic lethality in combination with the L35A point mutant than for the entire deletion of Not4 (36 versus 25 genes), and only 12 were in common [131]. 

Expression of a Not4 protein that does not interact with the other Ccr4-Not subunits, Not4_1–430_, grows essentially like wild-type cells at either 30 or 37°C on rich medium [82] (Table 1). This would suggest that under healthy favorable growth conditions, the association of Not4 with the other Ccr4-Not subunits is not important, consistent with the finding that the human protein is not in a stable complex with the other Ccr4-Not subunits [141]. However, cells expressing this truncated Not4 do not grow on media containing translational inhibitors, revealing that insertion of Not4 into the Ccr4-Not complex is important to overcome translation difficulties.

Several studies have tried to pinpoint the specific function of Not4. Microarray experiments have revealed that a limited number of genes, enriched for certain cellular functions, are affected by cells lacking Not4 growing in glucose at 30°C [143, 144]. These genes are clearly different from the genes deregulated when the other enzyme of the Ccr4-Not complex, namely the Ccr4 deadenylase, is deleted. In addition, while more genes are deregulated in the absence of Not4 than in the absence of Ccr4 under optimal growth conditions (467 genes versus 291), the situation is reversed when cells continue growing in the absence of glucose (680 genes versus 1429) [143]. One interesting observation is that upon glucose depletion from the growth medium Not4 is important to repress expression of genes encoding a number of ribosome-related proteins and translation initiation factors [143]. 

As mentioned above, another approach used to understand Not4 function has been a synthetic genetic array (SGA) approach, determining which nonessential yeast genes become essential in the absence of Not4 or when Not4 carried the L35A point mutation [131]. This approach led to the identification of genes involved in many different cellular functions, some of which will be discussed here. 

### 5.1. Not4 and Transcription

Many genes isolated in the SGA screen encode proteins connected to transcription [131]. This finding supported many early studies connecting the Ccr4-Not complex to the transcription machinery [125, 145–151] and more recent work, which showed that the Ccr4-Not complex can interact with RNA polymerase II and contribute to transcription elongation [152]. In addition, several proteins directly contributing to transcription or transcription regulation were identified as substrates for Not4. One such substrate is the Jhd2 demethylase that is polyubiquitinated by Not4 and targeted to degradation by the proteasome [153]. This enzyme removes methyl groups from histone H3 lysine 4, and consistently, in cells lacking Not4 when the demethylase accumulates, trimethylation of histone H3 lysine 4 is dramatically reduced [154, 155]. Levels of trimethylated histone H3 have been correlated with transcription levels; hence some of the described impact of Not4 on transcription in early studies might be related to its ubiquitination of Jhd2. In principle one expects that because Jhd2 acts in the nucleus, this is the cellular compartment where it is ubiquitinated by Not4. However, this expectancy is challenged by findings on a second-identified substrate of Not4, Cyclin C [156]. Cyclin C is a subunit of the cyclin-Cdk8 complex (also called the Srb11-Srb10 complex in yeast) that associates with the mediator, a regulator of transcription that associates with the RNA polymerase II holoenzyme. It is degraded by the proteasome in response to oxidative stress and is polyubiquitinated via lysine 48 linkages by Not4. Prior to degradation, it is translocated from the nucleus to the cytoplasm. Derivatives of cyclin C that are restricted to the cytoplasm are still degraded in response to oxidative stress in a Not4-dependent manner. Thus, ubiquitination of this nuclear transcription factor occurs in the cytoplasm. Inversely, another transcription factor Yap1 is translocated to the nucleus upon oxidative stress where it will not only activate its target genes, but also undergo accelerated degradation that depends upon its DNA binding. Oxidative stress increases Not4's interaction with Yap1, and Not4 is required for Yap1 degradation [157]. Taken together, it seems that Not4-dependent polyubiquitination of substrates occurs both in the cytoplasm and in the nucleus.

### 5.2. Not4 and the UPS System

The SGA screen with Not4 also identified many proteins involved in the ubiquitin-proteasome system. Amongst these, 2 genes, Doa4 and Ubp6, are required to maintain the pool of free ubiquitin in the cell, albeit for different reasons. Indeed, they encode de-ubiquitinating enzymes that recycle ubiquitin from proteins targeted, respectively, to the vacuole [158] and to the proteasome [159]. This led to the hypothesis that Not4 might also contribute to the pool of free-ubiquitin in the cell. Indeed, free ubiquitin levels drop in the absence of Not4 [82]. In addition, polyubiquitinated proteins accumulate in cells lacking Not4 [82], and Not4 interacts with proteasome subunits [154]. The sum of these observations hinted to a functional connection between Not4 and the proteasome.

The ubiquitin proteasome is the major machine that the cell has to degrade short-lived proteins [160]. It consists of a core particle (CP), which carries the catalytic enzymes, and a regulatory particle (RP), which recognizes ubiquitinated substrates, deubiquitinates them, unfolds them, and delivers them to the core particle. The core particle itself is composed of 2 rings of 7 *α* subunits and 2 rings of 7 *β* subunits with different hydrolytic activities. The regulatory particle is composed of a base, consisting of 9 subunits of which 6 are ATPases, and which functions to unfold substrates, open the channel to the core particle, and translocate the substrates to the core particle, and a lid composed of 8 non-ATPase subunits that serve for substrate recognition and deubiquitination. The phenotype observed in cells lacking Not4, namely, accumulation of polyubiquitinated proteins and reduced free ubiquitin, is compatible with inefficient proteasome function. Consistently, analysis of the proteasome in cells lacking Not4 reveals defects in proteasome integrity [82]. Two different pools of proteasomes are characterized in mutant cells. On one hand, some proteasomes are unstable and fall apart during isolation, leading to free active CP, but no detectable free RP, itself probably falling further apart. On the other hand some proteasomes are so tightly associated (probably incorrectly assembled) that they resist to high levels of salt. 

These findings indicate that Not4 contributes to assembly of the proteasome. The assembly of the proteasome has been well established [160–163] and can be reconstituted *in vitro* from core and regulatory particles, which can assemble separately with the help of specific chaperones, Pba1-4 and Ump1 for the core particle and Hsm3, Nas6, Nas2, and Rpn14 for the regulatory particle. Besides the well-defined chaperones that contribute to assembly of the individual particles, other proteins that associate with the proteasome and contribute to its stability, activity, and possibly also to its assembly have been identified. These include Ecm29 [164–168] and Blm10 [169–175]. Their exact function is unknown, but Ecm29 is thought to associate with, and inhibit, faulty proteasomes or to target the proteasome to specific cellular compartments, whereas Blm10 associates with RP-less CP to promote the degradation of proteasome substrates, either specifically or under specific conditions. 

The mechanism by which Not4 contributes to proteasome assembly has not been firmly established. The available evidence suggests that Not4 contributes to assembly of the regulatory particle. Indeed, in cells lacking Not4, regulatory particle that is not associated with core particle is unstable, despite the stability of the individual subunits. Furthermore some proteasomes are salt resistant, a faulty phenotype observed in the absence of Ecm29. Not4 interacts with proteasome subunits as well as with Ecm29, and the association of Ecm29 with proteasome subunits is altered in *not4Δ* mutant cells. Some of the mutant phenotypes of the proteasome observed in *not4Δ* are similar in cells lacking Ecm29, and aggravated in the double mutant (salt resistant proteasomes, growth on rich medium), whereas others are suppressed (unstable RP). Hence it seems that Not4 might interact with Ecm29 and proteasome subunits to allow their functional association. It is interesting to note that Duncan and Mata show in their paper revealing widespread cotranslational assembly of protein complexes, that in *S. Pombe* RP subunits associate with mRNAs encoding other RP subunits and Ecm29, in a polysome-dependent manner [16]. These observations suggest that Not4 might help cotranslational assembly of Ecm29 with RP subunits. If one considers the functions attributed to Ecm29 mentioned above, maybe Not4 then contributes to inhibition of faulty proteasomes via Ecm29, and/or targets proteasomes to specific compartments. 

The role of Not4 in proteasome integrity requires association of Not4 with the Ccr4-Not complex, but it does not require the E3 ligase activity of Not4 (see Table 1). In contrast, CHX sensitivity and lethality in combination with a deletion of Ubp6 are the phenotypes the most sensitive to mutations in Not4. Indeed, mutants of Not4 do not associate well with the other Ccr4-Not proteins (Not4_1–430_), but also the RING I64A mutant, display a very strong synthetic growth phenotype when combined with the deletions of Ubp6 and Doa4, and they do not grow on CHX, a phenotype common to all proteasome mutants [176]. Accumulation of polyubiquitinated proteins also occurs when the RING domain of Not4 is deleted or when Not4 carries the I64A point mutation, but it is less sensitive to loss of interaction of Not4 with other Ccr4-Not subunits. It requires a complete removal of the C-terminus of Not4 (Not4_1–232_) to be observed (see Table 1). These results show that the accumulation of polyubiquitinated proteins does not exactly follow the same Not4 requirements as proteasome integrity. Hence it may result from a combination of several defects. 

### 5.3. Not4 and mRNA Degradation

 As mentioned above, Not4 is associated in a complex with the major eukaryotic deadenylases. However not much is known about the contribution of Not4 to mRNA deadenylation by Ccr4 and Caf1. The rate of shortening of the poly(A) tail of a MFA2pG reporter mRNA largely dependent upon Ccr4 and Caf1 was reported to be only marginally reduced in cells lacking Not4 compared to wild-type cells [134]. No subsequent study has addressed a possible role of Not4 in deadenylation by Ccr4 and Caf1 in a systematic way. As mentioned above, microarray experiments have demonstrated that genes de-regulated in the absence of Not4 only very marginally overlap with genes deregulated in the absence of Ccr4 under 2 different growth conditions [143]. Obviously in such experiments one measures only the steady state level of mRNAs, to which synthesis as well as degradation contribute. Thus it is difficult to determine from these experiments how exactly the role of Ccr4 and Not4 differs with regard to mRNA degradation.

Furthermore, Not4 might impact on mRNA degradation beyond deadenylation. Indeed, degradation of mRNAs involves deadenylation, followed by decapping and 5′ to 3′ degradation via the Xrn1 exonuclease. Dhh1, a subunit of the decapping complex, is associated with the Ccr4-Not complex [177]. Whether Not4 has an impact on Dhh1 function is not known. The level of Dhh1 depends upon its association with the Ccr4-Not complex, but it binds a different region of the Not1 scaffold than Not4.

Finally, mRNAs are also degraded by the exosome, either in the nucleus during nuclear surveillance or in the cytoplasm under various circumstances. Several reports have shown that Not4 contributes to nuclear exosome function, namely in processing of snoRNAs [178] and Rrp6-dependent quality control of nuclear export [179], and overexpression of Not4 was reported to be toxic in nuclear export mutants [142]. If Not4 has an effect on the nuclear exosome, it could be that Not4 has an impact on the cytoplasmic exosome also.

### 5.4. Not4 and the Ribosome

A different approach was used to characterize the function of Not4, and this consisted in looking for stable proteins that might be ubiquitinated by Not4 [47, 51, 138]. This was done by comparing proteins ubiquitinated in wild-type cells to those ubiquitinated in cells lacking Not4. This study identified a ribosomal protein Rps7A and both subunits of the NAC ribosome-associated chaperone. These targets require Not4 for ubiquitination *in vivo* and are ubiquitinated by Not4 *in vitro*. 

Rps7A ubiquitination was not dependent upon any of the other nonessential subunits of the Ccr4-Not complex (Table 1). Quite the contrary, Rps7A ubiquitination was increased when cells expressed mutants of Not4 that were compromised for Not4's interaction with the Ccr4-Not complex (Not4_1–330_). Not4 mutant proteins (1–232) and to a lesser extent (1–180) carrying only the RNA recognition sequence and the RING domain were still able to ubiquitinate Rps7A, albeit weakly (Table 1). Mutation of the lysine residues ubiquitinated by Not4 in Rps7A did not reveal any particular phenotype. However, as for many yeast ribosomal proteins, Rps7A has a paralog, Rps7B, and expression of nonubiquitinated Rps7A in cells lacking Rps7B was lethal. Curiously Rps7B is not ubiquitinated by Not4 *in vitro* although all of the lysine residues are conserved in both paralogs, and the proteins are highly homologous. *In vivo* Rps7B is also much less ubiquitinated than Rps7A. These results suggest that Not4 has a different affinity for Rps7A than for Rps7B [138]. The 2 paralogs are most different in their N-terminal domains suggesting that this may be the region recognized by Not4. Interestingly this region of Rps7A in the ribosome seems to be accessible.

Ubiquitination of NAC allows its co-immunoprecipitation with Rpl25, and consistently Rpl25 does not coimmunoprecipitate with NAC*β* in the absence of Not4 [51], suggesting that Not4 contributes to association of NAC with the ribosome. Consistently, the 2 lysine residues ubiquitinated by Not4 are present in the first ribosome-binding sequence mapped for NAC*β* [45]. A GFP-NAC*α* fusion protein loses its diffuse cytoplasmic localization in the absence of Not4 and instead localizes to strange spots in the cytoplasm [47]. Intriguingly, this punctate localization requires the UBA domain of NAC*α*. Since aggregated proteins accumulate in cells lacking Not4 and NAC is present in the aggregates [138], one can imagine that these spots are aggregated ubiquitinated proteins to which NAC*α* is bound maybe via its UBA domain. This remains to be verified. It is interesting to note that NAC*α* copurifies with the proteasome and that this depends upon Not4 [51].

It has not been easy to pinpoint the exact role of the ubiquitination of these substrates by Not4, but these findings have revealed a cytoplasmic function for Not4, that could be further investigated. Not4, like all of the subunits of the Ccr4-Not complex, is associated with translating ribosomes [138]. The functional relevance of this association is supported by the reduction of polysomes when cells lack Not4 or other subunits of the Ccr4-Not complex. More importantly, aggregated proteins accumulate in *not4Δ*, and ribosomal proteins as well as NAC itself are present in these aggregates [138]. Very few proteins aggregate in the absence of NAC and polysome profiles appear normal. However, the deletion of NAC aggravates the accumulation of protein aggregates that accumulate in cells lacking Ssb/RAC, suggesting that nevertheless NAC contributes to protein solubility [58]. The role of Not4 ubiquitination of NAC in this context however is totally unclear. 

Aggregated proteins accumulate in the absence of Rps7A, but not Rps7B, and polysomes are altered. Why in the absence of Rps7A, rather than Rps7B, do aggregated proteins accumulate? Either they are produced in greater amounts in the absence of one of the 2 paralogs specifically or they are less efficiently removed. Maybe Rps7A is a better binding site for proteins that impact on cotranslational protein folding or for proteins which ubiquitinate misfolded nascent chains? However, this is unlikely because the relevant E3 ligase Ltn1 binds 60S and not 40S ribosomes (see above). Maybe Rps7A binds better proteins that contribute to clear ubiquitinated nascent chains or avoids their accumulation, be it proteasome subunits or the Ccr4-Not complex itself. Indeed, as mentioned above, there is evidence that Not4 has more affinity for Rps7A than for Rps7B. However, as for NAC, the role of the ubiquitination of Rps7A even in such a model is unclear.

### 5.5. Not4 and Quality Control

The finding that Not4 is present in translating polysomes, ubiquitinates a ribosome-associated chaperone and a ribosomal protein, both of which are important for protein solubility, and that aggregated proteins accumulate in cells lacking Not4 has very naturally led to the suggestion that Not4 contributes to cotranslational quality control. The very relevant question becomes what is Not4's exact contribution to this quality control, bearing in mind all that we have already learned about Not4. One first study [180] reported that nascent peptides provoking translation arrest accumulate in the absence of Not4 or in cells expressing the L35A point mutant, and the authors suggested that they might be ubiquitinated and degraded by Not4. However, we now know that Ltn1 ubiquitinates these peptides, leaving a possible role for Not4 in the degradation of the ubiquitinated peptides. It is known that these stalled peptides are degraded by the proteasome, but the inhibition of the proteasome did not aggravate the *not4Δ* mutant phenotype, suggesting that Not4 was involved in proteasome degradation of the arrested peptides. In this study the authors showed a concomitant increase in the no-go mRNA, suggesting also defective NGD in *not4Δ*. In another study [121], the deletion of Not4 was reported to only increase the level of a similar truncated nascent protein when the Ltn1 E3 ligase was deleted. Why was the deletion of Not4 sufficient to increase expression of a no-go protein in the first study but not in the second? It could be that this difference is due to the genetic background. Indeed, in the first study, the strain used was W303, whereas in the second study the background was BY4741. In BY4741 the deletion of Not4 does not lead to increases in a no-go protein, unless Ltn1 is deleted, suggesting that Not4 might come in to play only when some problem in the basic quality control occurs or when the stress put on the translation system increases. The presence of such a stress might be the difference between the 2 genetic backgrounds studied. Indeed, W303 is a widely used strain background in the yeast community, but it has amino acid alterations in 799 proteins, including factors of ageing and stress resistance, when compared to S288C, from which BY4741 was derived [181]. Significant differences between responses in these 2 genetic backgrounds have already been described [182]. It should be noted that in W303, most of Not4 was observed in polysomes [180], whereas only some Not4 was observed in polysomes in the BY4741 background [138]. Would the increased presence of Not4 in polysomes be indicative of the induction of a Not4 response? It would be interesting to determine whether in BY4741 cells lacking Ltn1, the amount of Not4 in translating ribosomes increases. 

So to conclude, it seems that Not4 contributes to cotranslational quality control, but it may not be amongst the first actors to come into play. It may serve rather in a second-surveillance step. Which then of the many functions attributed to Not4, are the ones participating to cotranslational quality control? 

#### 5.5.1. Transcription

 It seems unlikely that the transcription functions of Not4 are directly related to the cotranslational quality control. It is easy to imagine that changes in transcription levels can indirectly cause, improve, or worsen events at the ribosome, and Not4 might contribute to signaling from the ribosome in the cytoplasm to the transcription machinery in the nucleus to avoid producing excessive mRNAs when translation encounters problems. This idea however is rather theoretical at this time. 

#### 5.5.2. mRNA Degradation

 The increase of no-go mRNA in *not4Δ* indicates probably that at least part of the role of Not4 might be to signal the presence of translationally arrested peptides to the RNA degradation machine(s). The obvious target of Not4 is the deadenylase within the Ccr4-Not complex, and it seems likely that Not4 could induce activation of deadenylation to avoid accumulation of mRNAs that are translated into truncated and potentially toxic proteins. It is important to note that the deletion of Ccr4 leads to much less accumulation of ubiquitinated proteins when compared to the deletion of Not4 (unpublished observations). Thus, in any event it seems unlikely that the role of Not4 in protein quality control is limited to activation of the deadenylase within the Ccr4-Not complex. Since the Ccr4-Not complex has also been reported to interact with the exosome and Dhh1 of the decapping complex (see above), it remains possible that Not4 also communicates to the exosome and/or Dhh1 to accelerate degradation of the mRNA.

#### 5.5.3. Ubiquitination of NAC

 Not4 interacts with and ubiquitinates NAC, and this ubiquitination contributes to co-immunoprecipitation of NAC with L25. NAC and Ssb/RAC have partially overlapping functions, and as mentioned above, Ssb/RAC contributes to translation repression of nonstop and polylysine transcripts. However, it does not seem very likely that NAC contributes to translation repression in a Not4-promoted way. Indeed, upon NSD once the poly(A) tail starts to be translated, Ltn1 is still important to limit accumulation of the nonstop protein that needs to get degraded; Ssb/RAC is still required to maintain translational repression of nonstop transcripts, but Not4 does not contribute to limit accumulation of the nonstop protein [110, 121, 180]. Whether this is because in this case Pab1 is removed from the 3′ end and interaction with the translation initiation machinery is abrogated, whether it is because deadenylation is no longer relevant for mRNA degradation, or whether it is for some other reason, in any event, the role of Not4 is distinguishable from that of Ltn1 and Ssb/RAC. An attractive idea for the role of NAC ubiquitination by Not4 is that it might be relevant to the interaction of NAC with newly synthesized misfolded proteins that become ubiquitinated, and this might help them to be delivered to chaperones for additional folding and/or clearance by the proteasome. Indeed, Not4 improves the interaction of NAC with the ribosome and the proteasome, and NAC is found in the protein aggregates that accumulate in the absence of Not4. Furthermore NAC is present in cytoplasmic spots in the absence of Not4 in a UBA domain-dependent manner. Obviously more experiments are needed to address this model. 

#### 5.5.4. Ubiquitination of Rps7A

As mentioned above, Not4 can interact with Rps7A better than with Rpb7B, and aggregated proteins accumulate in cells lacking Rps7A but not lacking Rps7B. Hence if indeed these observations are relevant to cotranslational quality control by Not4, one can hypothesize that it is either the interaction of Not4 with Rps7A per se or ubiquitination of Rps7A by Not4 that is important to avoid accumulation of aggregated proteins. Regardless of which of these possibilities, if any is correct, the interesting aspect of Rps7A is its position in the ribosome. It lies in a region of the ribosome expected to be in close contact with the translation initiation machinery, in particular eiF3, and it stabilizes a large eukaryotic-specific cluster of ribosomal RNA called Expansion Segment 6 likely to participate in translation initiation ([183–185] and S. Melnikov personal communication). Thus, interaction of Not4 with Rps7A, or ubiquitination of Rps7A, might have an impact on translation. 

#### 5.5.5. Proteasome Assembly

 Inhibition of the proteasome does not increase the amount of translationally arrested polylysine protein that accumulates in W303 cells lacking Not4. It has been clearly demonstrated that these translationally arrested proteins are polyubiquitinated and cleared by the proteasome. Not4 does not prevent the ubiquitination of the peptide, which depends upon Ltn1. Hence one must conclude that the deletion of Not4 is contributing to the clearance of the ubiquitinated protein by the proteasome (though it may also reduce its production). At present our best model for how Not4 participates to proteasome degradation of translationally arrested polylysine protein is to propose that it improves assembly of the proteasome at the site of translation. Indeed, it is unlikely that the activity of the proteasome becomes globally limiting in cells lacking Not4, but local requirements for the proteasome might not be fulfilled. In support of this idea, a protein complex containing proteasomes and many components of the translation initiation machinery including ribosomal proteins and initial factors were identified and termed the translatome [186]. It should be noted that proteasome assembly is not measurably affected in cells expressing the L35A mutant of Not4, which nevertheless leads to accumulation of no-go proteins. Hence, either the E3 ligase activity of Not4 participates in sensing a problem during translation (unlikely) or it participates in transmission of the signal to effectors, and hence the proteasome cannot be the only target. Consistently, deletion of Not4 has a greater effect on accumulation of translationally arrested proteins than inhibition of the proteasome, suggesting that Not4 contributes to cotranslational quality control beyond proteasome function. It should be noted that a role of Not4 in proteasome function canot be tested in the context of an Ltn1 deletion. Indeed, in the absence of Ltn1, no-go proteins will not be ubiquitinated and hence will not be a target for the proteasome. Thus, in the absence of Ltn1, one is likely to observe effects of Not4 that are not connected to the proteasome.

#### 5.5.6. Signaling to and from Not4

We really have no idea as to what activates Not4 during cotranslational quality control or even whether it gets specifically recruited to translationally arrested polysomes. We do not know how Not4 is connected to the other players, such as Asc1, Dom34/Hbs1, and Ltn1. Furthermore, whatever the downstream effectors of Not4, the question of how Not4 transmits the signal is very unclear. Is this via allosteric or covalent modifications within the Ccr4-Not complex or via recruitment of proteins to the complex or release of factors from the complex? Translationally arrested polylysine proteins accumulate in cells expressing the L35A Not4 mutant. Hence it is certain that one way or another, the E3 ligase activity of Not4 is important in cotranslational quality control. Whether it is to sense the signal or to transmit the signal to effectors has to be determined. We know that there are many residues in Not4 that are phosphorylated. These include S92, S298, T300, T310, T312, S352, S542, and T543 identified in global studies [187, 188] as well as T334 and/or S342 present in an identified phosphopeptide [189]. Whether these modifications participate has to be tested. Not4 is also autoubiquitinated [138]. Autoubiquitination of Not4 could easily lead to allosteric changes within the Ccr4-Not complex or changes in protein interactions that could activate the deadenylase and other effectors. Such a model would explain why the L35A Not4 mutant is not able to perform its function in cotranslational quality control.

## 6. Perspective

**Figure 5:**
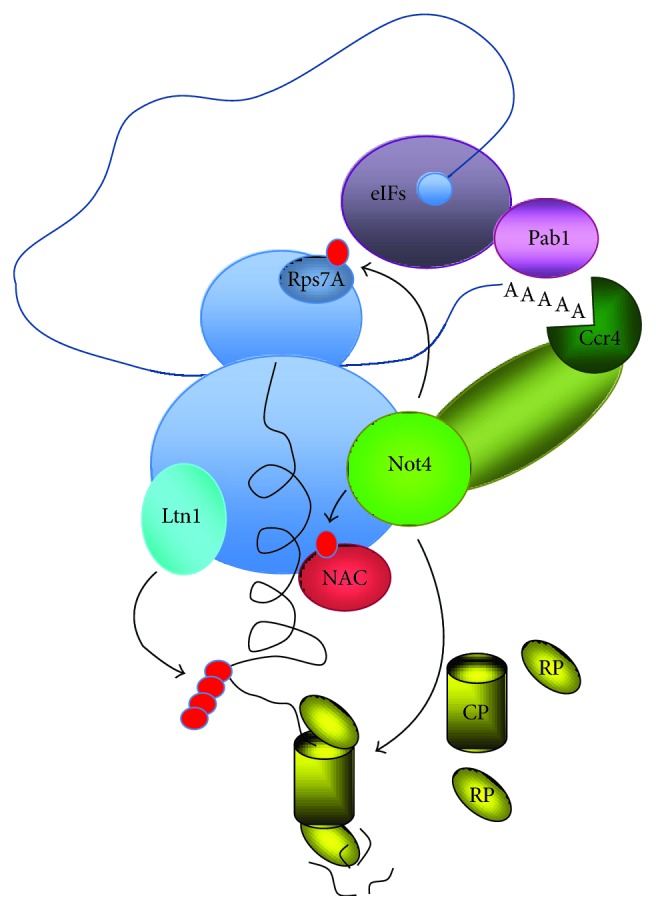
Model for the function of Not4 in quality control of newly synthesized proteins. After ribosome stalling in response to a problem in nascent protein folding or mRNA quality, the Ltn1 E3 ligase ubiquitinates the arrested peptide; Not4 is present at the ribosome and can favor proteasome assembly at the ribosome to degrade the ubiquitinated peptide and/or lead to activation of the Ccr4 deadenylase. Its ubiquitination of Rps7A in proximity to translation initiation factors might impact on translation repression, and its ubiquitination of NAC with its UBA domain might impact on the interaction of NAC with the ubiquitinated polypeptide that can be degraded by the proteasome, interact with other chaperones, or instead accumulate in protein aggregates.

Clearly we are still far from being able to integrate all that we know about Not4 to give a precise picture of its role in cotranslational quality control. What we can extract at this stage (Figure 5) is that Not4 can sense accumulation of problems during translation, in response to which it can act to prevent accumulation of aberrant proteins. Evidence linking Not4's response to the RNA degradation machines, certainly to the deadenylase (but maybe also the exosome and/or the decapping complex), and to the proteasome is available. Not4 also ubiquitinates NAC, stabilizing its interaction with the ribosomal protein L25 and the proteasome and potentially allowing it, via its UBA domain, to associate with the nascent chains that become ubiquitinated by Ltn1 and favor their interaction with the proteasome and/or other chaperones. Not4 also ubiquitinates Rps7A, that might lie in proximity to translation initiation factors and thereby somehow impact on translation. Many specific experiments can now be designed to tackle the great number of remaining questions about the role of Not4, taking advantage of the well-characterized point mutants and deletion mutants that are available, in combination with deletions of the different other components of the cotranslational quality control system. 

## Figures and Tables

**Table 1:** Summary of phenotypes measured for different Not4 derivatives.

Phenotypes/Not4 derivatives	1–587	1–430	1–180	78–587	164A	L35A
Growth 30°C (doubling time in hrs in YPD)°	2.5	3.5	4.6	5	3.1	nd
Growth 37°C (YPD)°	Yes	Yes	No	No	Yes	Yes
Growth on CHX°	Yes	No	No	No	Yes	nd
Hydroxyurea or MMS^&^	Yes	nd	nd	nd	nd	No
Resistance to heat stress^%^	No	nd	nd	nd	Yes	Yes
SL or SS with *ubp*6 and/or *doa*4°	No	ss	sl	sl	ss	nd
Accumulation of polyubi°	No	No	Yes	Yes	Yes	nd
Unstable RP°	No	Yes	nd	Yes	No	nd
Salt-resistant RP-CP°	No	Yes	nd	Yes	No	nd
Ubiquitination of Rps7A∗	Yes	Yes	Yes	No	No	nd
Toxic when overexpressed in *hpr*1 or *nup*116^+^	Yes	nd	nd	nd	nd	Yes
Binding to Ccr4-Not°	Yes	Weak	nd	Yes	Yes	nd

The phenotypes measured for different derivatives or Not4 in different studies: °[82], ^&^[140], ^%^[131], ∗[138], and ^+^[142] are summarized here. Growth on different media or when combined with different mutants is indicated by yes or no and or by the doubling time in hours or finally by sl (synthetically lethal) or ss (synthetically sick). nd, not determined.
